# Diamond compound refractive lenses for high-energy dark field X-ray microscopy

**DOI:** 10.1107/S1600577526009859

**Published:** 2026-10-29

**Authors:** S. Staeck, C. Yildirim, F. Seiboth, T. Manning, T. Roth, J. C. Stinville, C. Detlefs

**Affiliations:** a European Synchrotron Radiation Facility Grenoble France; b Technical University of Denmark Department of Physics Kgs Lyngby Denmark; c Centre for X-ray and Nano Science CXNS Deutsches Elektronen-Synchrotron DESY Hamburg Germany; d Materials Science and Engineering Department University of Illinois Urbana-Champaign Urbana USA; Tohoku University, Japan

**Keywords:** CRL, diamond, DFXM, X-ray optics

## Abstract

Compound refractive lenses (CRLs) are a type of X-ray optics that find widespread applications as focusing and imaging lenses. The choice of material is one of the most defining properties of these lenses. In this work, we present a CRL made out of diamond. It provides an advantageous balance between refractivity and absorption, along with good manufacturability. Compared with Be CRLs, it features a higher optical density and thus enables application at higher photon energies without relying on large lens stacks or very small radii of curvature, which are challenging to manufacture. A diamond CRL is characterized for use as an objective for dark-field X-ray microscopy (DFXM) at the ID03 beamline of the European Synchrotron Radiation Facility and compared with Al and Be CRLs at 17 keV, 33 keV and 37 keV. Increasing the photon energy in DFXM from 17 keV up to 37 keV opens up the possibility to investigate new sample systems that were previously opaque to low-energy X-ray radiation. The capability of the diamond CRL at 33 keV is illustrated through DFXM measurements on two 0.5 mm-thick iron-based samples, which cannot be probed at 17 keV.

## Abstract 2

In this work, we compare and quantify the performance of compound refractive lenses fabricated from single-crystal diamond against conventional beryllium and aluminium counterparts under bright-field conditions and show dark-field imaging applications for the diamond compound refractive lens.

## 1. Introduction

The concept of using compound refractive lenses (CRLs) to focus X-rays was first introduced in a 1994 patent by Tomie (Tomie, 1994 ▸). Tomie proposed that, despite the extremely low refractive indices of materials at X-ray wavelengths, effective focusing could be achieved through a series of concave lenses fabricated from low-atomic-number (low-*Z*) materials. This transmissive lens-based approach stood in contrast to established methods relying on grazing incidence reflection or diffractive optics such as Fresnel zone plates.

In 1996, Snigirev *et al.* demonstrated a practical implementation of CRLs at a synchrotron radiation source (Snigirev *et al.*, 1996 ▸). By drilling cylindrical holes into an aluminium block, they created the first working prototype of a refractive X-ray lens, thereby establishing the feasibility of compact, in-line X-ray focusing optics for high-energy applications. Since then, CRLs have become a widely adopted solution in synchrotron and XFEL beamlines, used in diverse applications such as phase-contrast imaging, tomography, full-field microscopy, and nano-focused X-ray diffraction (Lengeler *et al.*, 1999 ▸).

Manufacturing advances have greatly improved the optical quality of CRLs, enabling sub-micrometre precision and reduced aberrations. Techniques such as deformation via indentation, deep X-ray lithography, LIGA processing, laser micromachining, and 3D nanoprinting now allow the fabrication of lenses from a broad range of materials (Roth *et al.*, 2017 ▸). These developments have expanded the design space to include transfocators for focal length adjustment (Snigirev *et al.*, 2009 ▸; Vaughan *et al.*, 2011 ▸), beam-shaping geometries like alligator lenses (Cederström *et al.*, 2000 ▸), and CRLs made from advanced polymers such as SU-8 (Krywka *et al.*, 2016 ▸).

**Figure 1:**
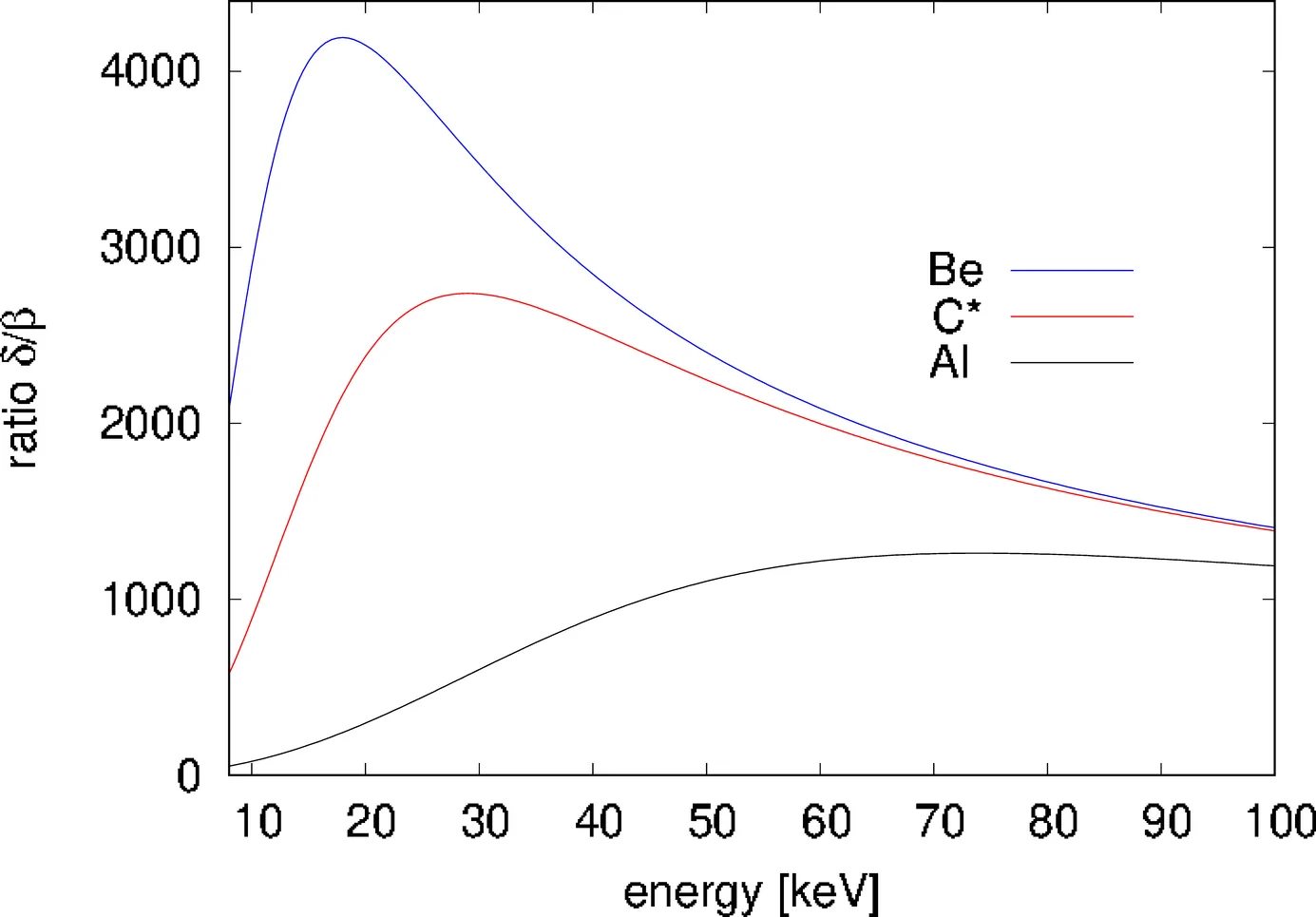
Ratio of δ/β for the three most commonly used lens materials.

Material selection is a key factor in CRL design, as both focusing efficiency and X-ray transmission are determined by the complex refractive index *n* = 1 − δ + *i*β. The real part, δ, governs the lens’ focusing power, while the imaginary part, β, describes absorption. For efficient lens systems, especially those with many elements, it is crucial to maximize δ while minimizing β to preserve beam intensity. Fig. 1 ▸ shows the ratio δ/β for Al, Be and diamond. Among available materials, beryllium (Be) has long been considered ideal for hard X-ray applications due to its favorable combination of high transmission and sufficient focusing strength (Serebrennikov *et al.*, 2016 ▸). Be CRLs have been successfully deployed in both condenser and objective roles, including at the ESRF’s ID06-HXM beamline for dark-field X-ray microscopy (DFXM; Kutsal *et al.*, 2019 ▸), now upgraded as ID03 (Isern *et al.*, 2025 ▸), and in X-ray free-electron laser (XFEL) facilities (Heimann *et al.*, 2016 ▸; Zozulya *et al.*, 2019 ▸; Dresselhaus-Marais *et al.*, 2023 ▸).

Despite these advantages, the practical use of Be is increasingly constrained. The material is toxic, brittle, and expensive, with growing difficulties in procurement. Moreover, structural imperfections such as voids, inclusions, and grain boundaries can introduce small-angle scattering, degrading coherence and image quality (Timmann *et al.*, 2009 ▸; Roth *et al.*, 2014 ▸; Lyatun *et al.*, 2020 ▸). These issues have led to the discontinuation of commercial Be lens production, for instance, by RXOPTICS, accelerating the search for alternative materials better suited to modern synchrotron and XFEL environments.

Aluminium lenses are relatively cheap and easy to manufacture and therefore display good imaging performance with small lens errors (Snigirev *et al.*, 1998 ▸; Schroer *et al.*, 2000 ▸). They are also relatively stable under thermal load in high-flux applications. However, diamond and beryllium offer better transmission, especially at lower energies (below 20 keV to 30 keV).

Nickel CRLs offer a robust option for focusing high-energy X-rays (Nazmov *et al.*, 2005 ▸; Andrejczuk *et al.*, 2014 ▸; Brancewicz *et al.*, 2016 ▸). Since Ni is a rather heavy element, it has a high diffractive power combined with high absorption losses. Therefore, it is mainly applicable at high energies (>30 keV), where the absorption losses are tolerable.

Silicon is a radiation-hard material with excellent thermal and mechanical stability, increasingly used for CRLs in hard X-ray optics (Schroer *et al.*, 2003 ▸; Schroer & Lengeler, 2005 ▸; Simons *et al.*, 2016 ▸; Ruett *et al.*, 2022 ▸). Again, compared with Be or diamond lenses, the diffraction to absorption ratio is smaller, especially at lower energies.

Polymeric CRLs fabricated from SU-8 negative photoresist offer a practical solution for X-ray focusing in the 5 keV to 30 keV range (Marschall *et al.*, 2014 ▸; Simons *et al.*, 2015 ▸; Yildirim *et al.*, 2020 ▸; Hlushko *et al.*, 2020 ▸). They are comparatively easy and reproducible to manufacture and feature low absorption, but lack radiation hardness and thermal stability.

Among all candidates, diamond has emerged as the most likely material for next-generation CRLs. It combines a relatively low β (high transmission) with high δ (strong focusing) in the hard X-ray regime. Most importantly, its superior thermal conductivity makes diamond lenses uniquely suited for high-heat load applications, such as white and pink beam transfocators or XFEL optics (Antipov *et al.*, 2016*a* ▸; Isern *et al.*, 2025 ▸; Yildirim *et al.*, 2025 ▸; La Bella *et al.*, 2025 ▸; Dresselhaus-Marais *et al.*, 2023 ▸; Irvine *et al.*, 2025 ▸). The intrinsic radiation hardness and structural integrity of diamond allow stable performance under extreme flux conditions. The single-crystal substrates used for manufacturing diamond CRLs have excellent homogeneity, therefore exhibit much lower small-angle scattering than polycrystalline Be, especially when the optical surfaces are polished (Timmann *et al.*, 2009 ▸; Gasilov *et al.*, 2017 ▸). Even if the lenses are not polished, they exhibit a similar or lower scattering intensity than comparable polycrystalline Be lenses in small-angle X-ray scattering (SAXS) (Celestre *et al.*, 2022 ▸). Despite fabrication challenges, requiring focused ion beam (FIB) milling or laser micromachining, recent advances have demonstrated reliable production of diamond CRLs with excellent optical quality (Snigirev *et al.*, 2002 ▸; Antipov *et al.*, 2016*b* ▸; Polikarpov *et al.*, 2016 ▸; Seiboth *et al.*, 2017 ▸; Celestre *et al.*, 2022 ▸; Wang *et al.*, 2025 ▸; Wang *et al.*, 2026 ▸).

In this work, we compare and quantify the performance of CRLs fabricated from single-crystal diamond against conventional beryllium and aluminium counterparts under bright-field conditions and show dark-field imaging applications for the diamond CRL. Using a systematic set of test measurements, we assess resolution, field of view and transmission efficiency across a range of photon energies from 17 keV to 37 keV. Diamond CRLs were benchmarked against Be CRLs at 17 keV and Al CRLs at 33 keV, using standardized JIMA resolution targets and Si checkerboards to characterize optical quality and aberrations. Additionally, DFXM measurements at 33 keV were performed on recrystallized ferritic iron and Invar alloy samples to evaluate the suitability of diamond CRLs for high-resolution diffraction contrast imaging, including mosaicity mapping. This comprehensive dataset provides a practical basis for selecting CRL materials for high-flux synchrotron environments and emerging fourth-generation light sources. We show that diamond is a suitable material for CRL manufacturing to replace barely available and toxic Be CRLs. Furthermore, the higher refractive power of diamond CRLs compared with Be CRLs enables existing X-ray microscopes to operate at higher photon energies, thereby extending the usable energy range of the DFXM setup at ID03 in particular. At ID03, the range of applicable samples can be extended to heavier and thicker samples.

The following sections describe the experimental configurations, imaging modalities, and evaluation metrics used in this comparative study.

## 2. Experimental setup

Different types of CRLs were characterized in terms of their resolving power at different energies, the extent of the spatial distortion and their field of view (FOV). Furthermore, two application samples were investigated to showcase the potential of the diamond CRLs for DFXM in Section 3.3. All these measurements were performed at beamline ID03 of the European Synchrotron Radiation Facility (ESRF), which is described in the following subsection. The characterization results of the different CRLs are presented below.

### 2.1. Lens stacks

Four CRL stacks have been tested in bright-field X-ray microscopy:

(i) At 17 keV, an *N* = 20, *R* = 25 µm diamond lens stack and an equivalent *N* = 88, *R* = 50 µm Be lens stack.

(ii) At 33 keV, an *N* = 124, *R* = 30 µm Al lens stack and an equivalent *N* = 70, *R* = 25 µm diamond lens stack.

(iii) The *N* = 70 diamond CRL was also used for dark-field X-ray microscopy, both at 33 keV and at 37 keV.

Table 1 ▸ summarizes the geometrical dimensions of the lens stacks. In the following figures and tables, the symbol C* refers to the diamond CRL.

The diamond lens stacks were produced at DESY using femtosecond laser ablation machining into single-crystalline chemical vapor deposited (scCVD) diamond plates (Wang *et al.*, 2025 ▸) on either side, leading to bi-concave lenses. No polishing step was applied after the laser-ablation. The surface micro-roughness after the ablation is typically below 200 nm (RMS). As transmission through diamond is high, especially at 33 keV, a tungsten pinhole of 120 µm diameter, slightly smaller than the diamond lens physical aperture, was used to reduce unwanted background from X-rays passing by the side of the physical lens aperture. After every ten lens plates, a previously determined correction optics phase plate was added to the stack in order to compensate for fabrication errors (Seiboth *et al.*, 2017 ▸). For the high-energy lens stack, this meant seven correction plates. The benefit of distributing many correction plates along the lens stack is that such a corrected lens stack can be used over a wide X-ray energy range without deteriorating the quality of the correction (Wang *et al.*, 2025 ▸). In this publication, the Strehl ratio could be improved from 0.26 to 0.76 using two phase plates for a diamond lens stack consisting of 60 lenses at 17 keV.

The Be lens stack was assembled using Be lenses purchased from RXOPTICS, with a mixture of Be grades coming from powder metallurgy and others from rolled ingot grades (Dombrowski, 1997 ▸; Roth *et al.*, 2014 ▸). The latter show less SAXS background, but, owing to the high number of required lenses, we were forced to use a mixture, as currently no lenses with rolled ingot grades are commercialized.

The Al lens stack was produced at the ESRF using 99.999% pure aluminium disks of 4 mm diameter and 1 mm thickness, polished to 40 nm r.m.s. surface roughness and pre-thinned to 600 µm, using flat punches. As with the Be lenses, the parabolic lens shape is obtained via a deformation process using two punches, in our case with parabolic *R* = 30 µm apex-radius tips, made in hardened steel. The width at the apex was 15 µm on average.

### 2.2. Beamline setup

**Figure 2:**
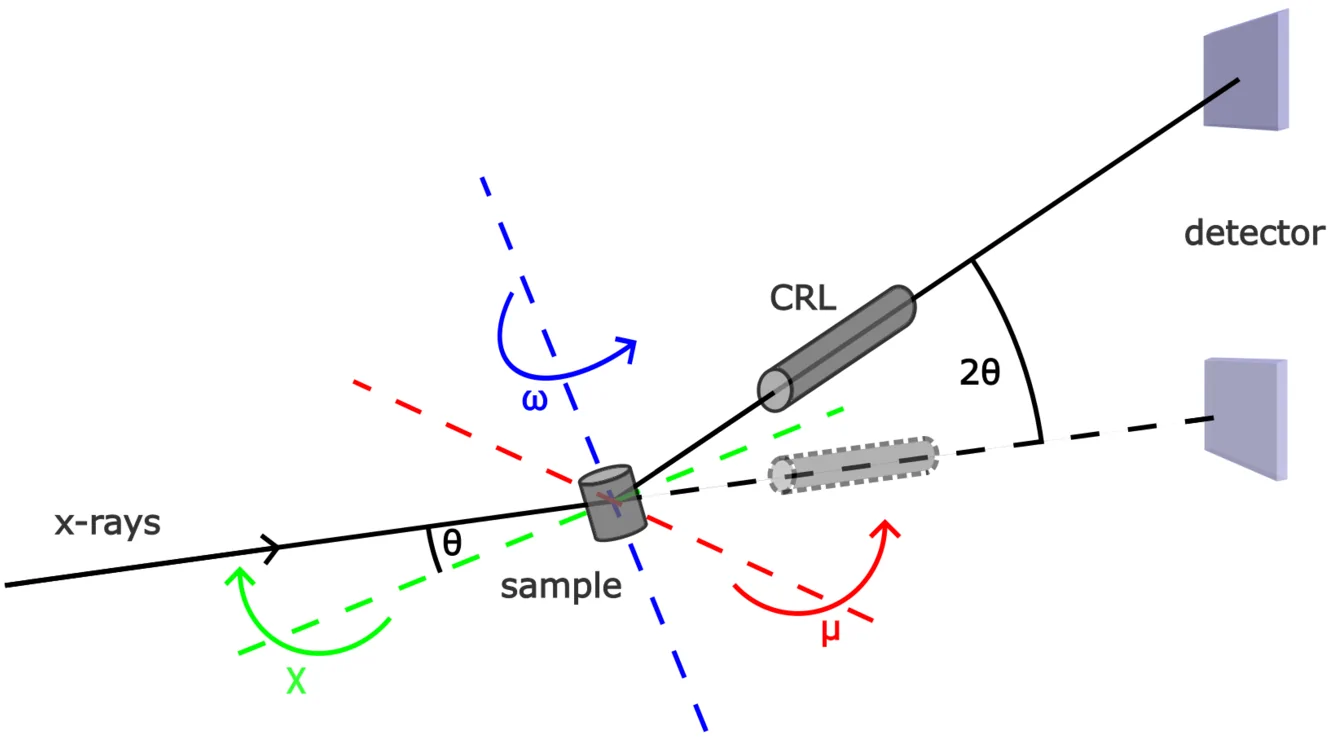
Schematic of the DFXM setup at the ID03 beamline at the ESRF. Depicted is the basic setup geometry with the dark-field (solid line) and bright-field (dashed line) configuration. In dark-field mode, the incoming beam is diffracted at the sample and the diffracting part of the sample is imaged by the CRL on the detector. Here, θ describes the Bragg angle. The CRL objective and the detector are positioned at an angle of 2θ with respect to the optical axis. μ, χ and ω are depicted as well. μ and χ can be used to investigate the mosaicity of a sample by bringing misoriented parts of the sample into diffraction condition. In bright-field mode, the CRL and the detector are in line with the incoming X-ray beam and the sample is imaged via absorption and phase contrast.

The ID03 beamline at ESRF (Isern *et al.*, 2025 ▸) specializes in dark-field X-ray microscopy, DFXM. This technique utilizes X-ray optics in the Bragg-diffracted beam to image the crystalline microstructure of samples. Using the diffraction contrast, the technique is sensitive to the orientation (mosaicity) and *d*-spacing (strain) of lattice planes in the sample (Simons *et al.*, 2015 ▸; Poulsen *et al.*, 2017 ▸). A schematic of DFXM at the ID03 beamline is shown in Fig. 2 ▸.

The beamline utilizes a multilayer and a crystal monochromator to monochromatize the X-ray beam generated by a cryogenic permanent magnet in-vacuum undulator. The beam is shaped by several sets of slits before it hits the sample. ID03 has an energy range from 11 keV to 60 keV; DFXM experiments are usually performed at 17 keV or 19 keV. A 1D Be CRL condenser may be used to generate a sheet beam for layered measurements of the sample (section topography). All measurements presented were performed with a box beam. The sample is mounted on a hexapod, which is again mounted on a goniometer for sample alignment and measurement. The goniometer features two axes of translation and two axes of rotation, μ and ω, see Fig. 2 ▸. The magnified image is recorded by a far-field detector at a distance of 2.5 m to 5 m downstream of the sample. This is an indirect detector comprising a crystal scintillator, magnifying optics (×2 or ×10), and an sCMOS camera (pco.Edge 4.2BI).

The goniometer angles μ and χ can be used to investigate the mosaicity of a sample to bring different, slightly mis­oriented sample parts into diffraction condition. The strain of a sample can be investigated by either scanning the scattering angle 2θ by moving the objective and far-field detector, or by scanning the photon energy. Such scans are typically combined with rocking scans (μ). Further experimental details are given by Isern *et al.* (2025 ▸).

In the measurements presented here, the sample-to-detector distance was 5 m. The values for magnification, effective pixel size and exposure time are given in the respective subsections. In general, the magnification is around *M* = 140–150, including the magnification of the ×10 far-field objective, and the effective pixel size is typically around 40 nm to 50 nm. The exposure time was either 1 s or 2 s, depending on the measurement.

## 3. Results and discussion

In this section, the results will be presented and discussed.

### 3.1. Bright-field X-ray microscopy

This subsection presents the resolution, radial distortion, and FOV in bright-field (2θ = 0).

#### 3.1.1. Resolution

**Figure 3:**
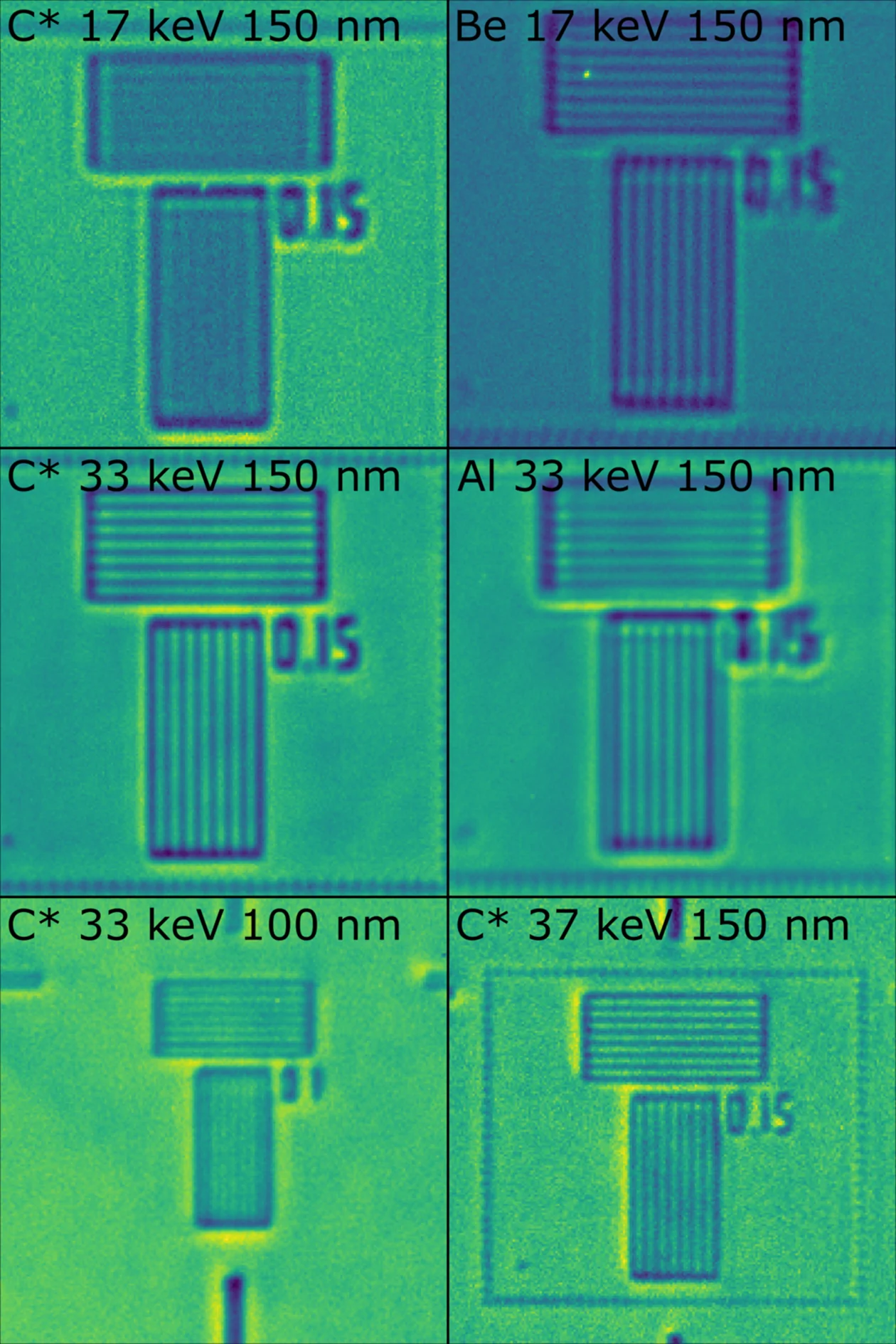
JIMA test pattern structures for different combinations of CRL objectives, X-ray energies, and structure sizes.

The resolving power of the diamond CRL was tested at different energies using a JIMA test pattern (JIMA, 2025 ▸). The results are compared with those of the Be CRL currently in use at low energies (17 keV) and with an Al CRL at higher energies (33 keV). Furthermore, test patterns have been recorded with the diamond CRL at 37 keV to investigate the potential to use this lens at even higher energies. The diamond lens stack has been adjusted according to the energy as presented in Table 1 ▸. For the lower-energy experiment, the energy in the case of the diamond CRL was slightly altered to 17.7 keV, to keep a similar focal distance and magnification as for the Be CRL. We will refer to this energy as 17 keV from here on as well, for the sake of simplicity. For the measurements, the 100 nm, 150 nm and 200 nm structure size test patterns on the JIMA resolution target were imaged in bright-field with the pco far-field detector and the 10× objective. From the images the Michelson contrast was calculated in the vertical and horizontal direction. The results are presented in Table 2 ▸, together with the maximum contrast measured in the images on a large structure on the JIMA resolution target. A choice of images of the JIMA test pattern for different combinations of CRLs, photon energies and structure sizes can be seen in Fig. 3 ▸. The values for magnification and effective pixel size for the far-field detector with the 10× objective and exposure time of the characterization measurements are given in Table 3 ▸. The magnification includes the magnification from the objective. For the 2× objective, the values for magnification and effective pixel size shown in Table 3 ▸ scale with a factor of five, accordingly.

At 33 keV, the diamond CRL achieves better contrast and resolution than the Al CRL, while at 17 keV the Be CRL provides superior resolution compared with the diamond CRL. This ordering follows directly from the numerical apertures (NAs) of the three-lens systems, with NA_Be_ > 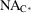 at 17 keV and 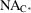 > NA_Al_ at 33 keV. At 17 keV, the Be CRL reaches NA_Be_ = 5.47 × 10^−4^, more than twice that of the diamond CRL (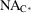 = 2.25 × 10^−4^), which accounts for both the resolution and the contrast difference observed at this energy. For the Al CRL the effective aperture *D*_eff_ is set by absorption, whereas for the diamond CRL it is limited instead by the 120 µm pinhole placed in front of the lens stack. The Be CRL is aperture-limited as well. The actual aperture of the diamond CRL is 140 µm. The pinhole covers the outer part of the aperture, where the optical properties suffer from manufacturing limitations. Although without the pinhole a theoretical NA of 2.55 × 10^−4^ (Lukas, 2026 ▸; Lengeler *et al.*, 1999 ▸) could be achieved, the resolution would degrade practically. At 33 keV, the diamond CRL reaches 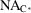 = 2.02 × 10^−4^, exceeding that of the Al lenses (NA_Al_ ≃ 1.5 × 10^−4^), and explains the gain in both contrast and resolution. Here, the NA of the Al CRL is absorption-limited in comparison with the diamond CRL, where the NA is aperture-limited. Consistently, the diamond CRL at 33 keV resolves 100 nm structures with a quality comparable with that achieved at 150 nm at 17 keV. Furthermore, as shown in Fig. 3 ▸ (bottom right), the diamond CRL is capable of imaging 150 nm structures even at 37 keV.

#### 3.1.2. Radial distortion and FOV

In an effort to characterize radial distortions (pin-cushion or barrel) of the diamond and the Al CRLs at 33 keV and the diamond and the Be CRLs at 17 keV, a 10 µm checkerboard pattern was imaged in direct beam geometry. Previously tested diamond CRLs exhibited serious radial distortion. For this reason, the new diamond CRL together with the existing CRLs were subjected to these measurements. Since radial distortions can heavily affect the image recorded with any microscope, it is important to quantify this effect, so that, if present, it can be corrected in data processing. With this, the distortion of the straight lines of the pattern could be observed. The radial distortion is quantified by applying the first order of the Brown–Conrady radial distortion model (Brown, 1966 ▸; Conrady, 1919 ▸),
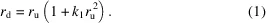
Here, *r*_d_ is the distorted distance from the pixel in question to the distortion center (which is assumed to be the center of the intensity distribution), *r*_u_ is the undistorted distance and *k*_1_ is the parameter describing the direction and magnitude of the radial distortion. For *k*_1_ < 0, *r*_d_ is smaller than *r*_u_ and the distorted pixels are shifted towards the distortion center. In this case, the image experiences a barrel distortion. Likewise, for *k*_1_ > 0 the image experiences a pin-cushion distortion. A detailed derivation of the *k*_1_ parameter from the checkerboard test sample images can be found in Appendix *A*[App appa].

**Figure 4:**
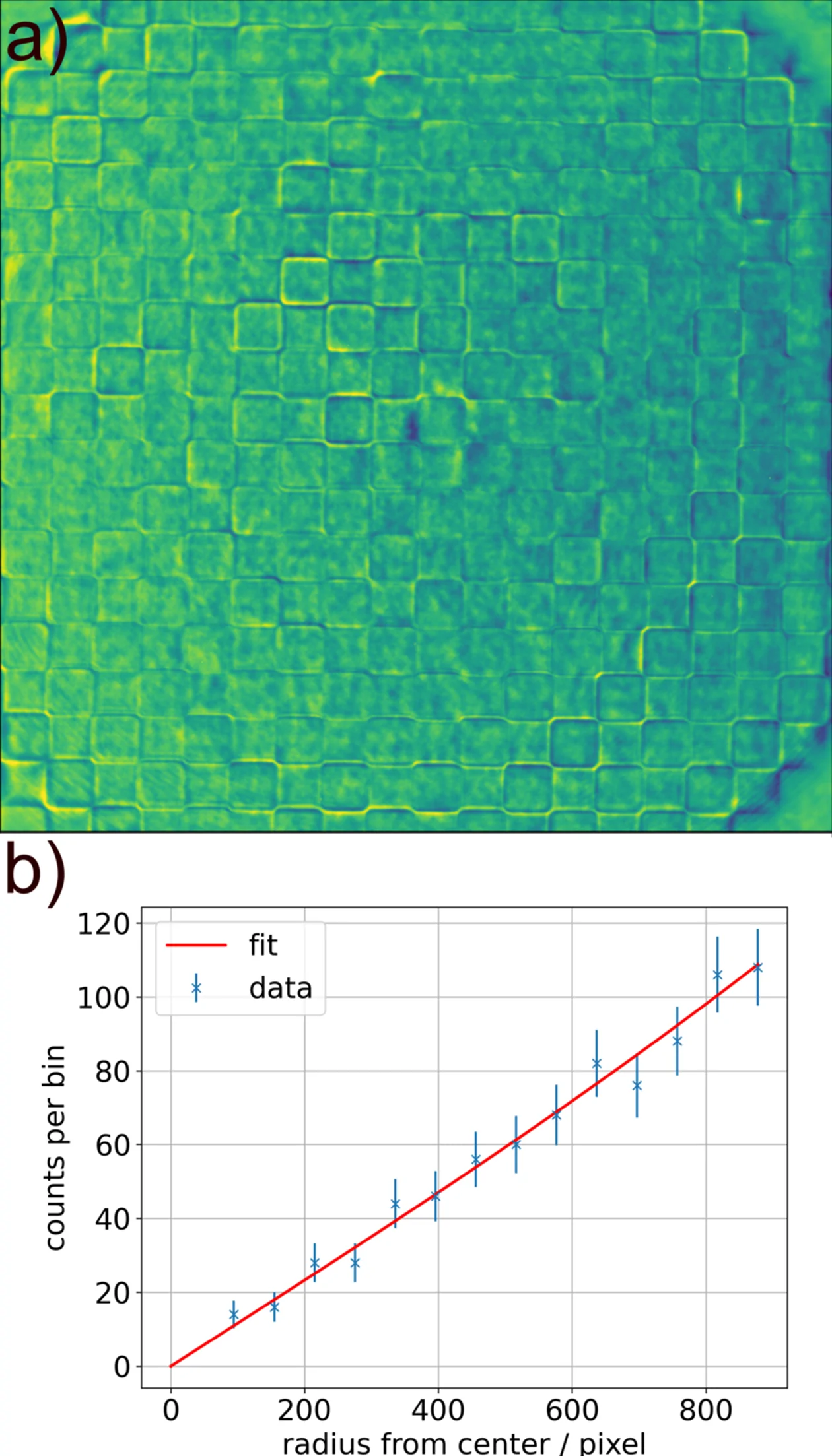
(*a*) Checkerboard pattern imaged with the diamond CRL at 33 keV. (*b*) Fit of the edge points per histogram bin in dependence on the radius from the center of mass of the intensity distribution of (*a*). The error bars represent the statistical uncertainties. A slight deviation from the linear relation is visible.

The checkerboard pattern with its known cell size of 10 µm is used as a scale for the calibration of the pixel size. For this, a cell in the center of the FOV is chosen, because there should be no radial distortion present. In addition to that, the measurement of the checkerboard pattern is used to estimate the FOV of the different objectives at different energies by calculating the full width at half-maximum (FWHM) of the intensity distribution. The effective pixel size is given as well. The FOV values are averaged over the *x*- and *y*-direction of the image. Note that the measurements for 33 keV were performed with the 10× objective on the pco far-field camera in comparison with the 2× objective for the measurements at 17 keV. The *k*_1_ values are given in µm^−2^ for comparability. An examplary checkerboard image recorded with the diamond CRL at 33 keV and the fit of the histogram of edge points are shown in Fig. 4 ▸. The results are shown in Table 4 ▸.

The results show small values for *k*_1_. Pin-cushion distortions are noticeable for all lenses, although negative *k*_1_ are within the uncertainty budgets for the lenses at 33 keV. The uncertainties are quite high, which shows that the measurement method might not be suitable for quantifying radial distortions that small. For example, *k*_1_ = 1.3 × 10^−5^ µm^−2^ for the Be lenses at 17 keV induces a shift of about 960 nm at a radius of 42 µm, which corresponds to a third of the respective FOV. For the lenses at 33 keV, the effect seems to be negligible, considering the high uncertainty budgets. Especially for the Be CRL at 17 keV, the effect should be further investigated and post-processing algorithms should possibly be applied. The FOVs differ rather drastically for different lenses, with the Be lenses exhibiting the largest FOV with 125.11 µm. The diamond lenses have a FOV of around 90 µm at both 17 keV as well as 33 keV, while the Al lenses have the smallest FOV with 64.55 µm.

### 3.2. Characterization results

The findings of the previous sections regarding the characterization of the different CRLs at different energies are presented in Table 5 ▸. The results for the contrast are given for the 150 nm resolution target (averaged over both spatial directions) as well as the results for the FOVs and the theoretical NAs.

### 3.3. High-energy dark-field X-ray microscopy

High-energy DFXM measurements above 30 keV have previously been reported only for a few cases using SU-8 CRL objectives at the ESRF ID06-HXM beamline, the former home of the Hard X-ray Microscope at ESRF (Kutsal *et al.*, 2019 ▸; Hlushko *et al.*, 2020 ▸; Yildirim *et al.*, 2022 ▸). Building on those demonstrations, the new diamond CRL objective was utilized here in two experiments showcasing the potential of diamond optics for high-energy DFXM. At 33 keV, two different iron-based samples were investigated. Operation at 33 keV within the geometrical constraints of ID03 enables access to sample systems that are strongly attenuating at 19 keV and below, thereby extending DFXM to thicker specimens and to compositions containing heavier elements. The results are presented in the following subsections.

#### 3.3.1. Recrystallized iron

The iron sample investigated here was fully recrystallized high-purity iron and is similar to the material studied by Shukla *et al.* (2025 ▸) and Sanna *et al.* (2026 ▸), where full details of the sample preparation and characterization are provided. Briefly, the material was cold rolled to a 90% thickness reduction and subsequently annealed at 700°C for 30 min to achieve a fully recrystallized microstructure. A needle-shaped specimen was extracted from the rolled plate by electrical discharge machining and electro polished to remove surface damage. DFXM measurements were performed near the top section of the sample within a local cylindrical volume of approximately 500 µm in diameter, where the average grain size was 15 µm to 20 µm.

**Figure 5:**
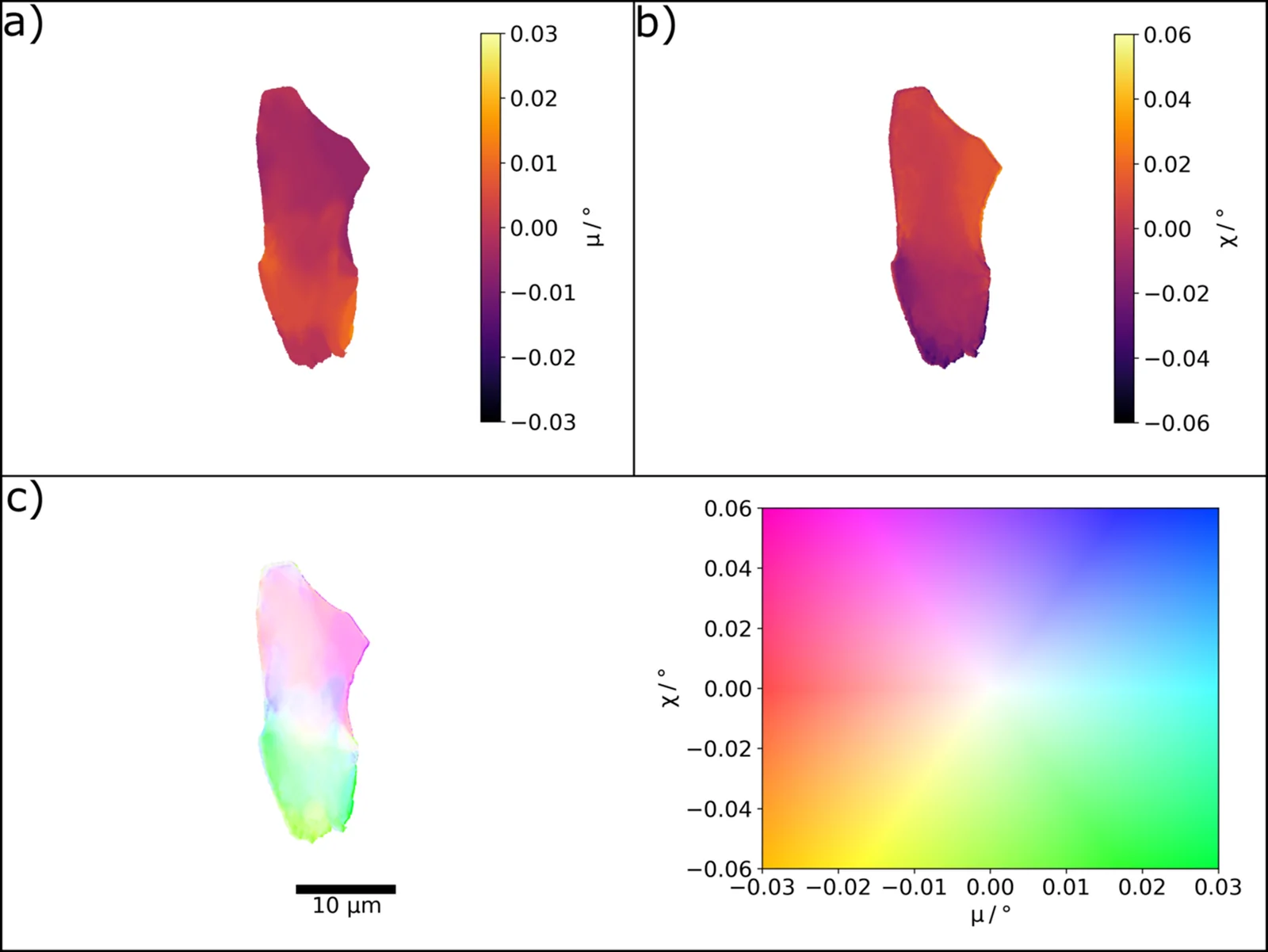
Grain 1 of the recrystallized iron sample imaged with the diamond CRL objective at 33 keV. (*a*) Rocking angle μ at maximum intensity for each pixel. (*b*) Rolling angle χ at maximum intensity for each pixel. (*c*) Combined intragranular misorientation map referenced to the center of the μ and χ rocking curves, revealing local lattice rotations associated with intragranular defects and elastic strain heterogeneities. The colormap and scale bar are indicated.

Figs. 5 ▸ and 6 ▸ show mosaicity maps of two representative grains acquired using the diamond CRL objective at 33 keV, demonstrating the suitability of the optics for high-energy DFXM. The magnification was *M* = 32.5 with the 2× far-field objective and the effective pixel size was 200.0 nm.

The intragranular misorientation maps of the recrystallized iron grains show a narrow angular spread, as expected for fully recrystallized high-purity iron with a low dislocation density on the order of 10^11^–10^12^ m^−2^ (Sanna *et al.*, 2026 ▸). Over most of the grain interior, both the μ- and χ-dependent maps exhibit only weak orientation variations, indicating a low level of stored lattice curvature.

In contrast, increased intragranular misorientation is observed in the vicinity of the grain boundaries in both the μ and χ maps. These regions of enhanced angular spread reflect local lattice rotations induced by mechanical constraint and compatibility stresses arising from the interaction with neighboring grains. The combined misorientation maps in Figs. 5 ▸ and 6 ▸ highlight this behavior, with the largest deviations concentrated near the grain edges, while the grain interior remains comparatively uniform.

#### 3.3.2. Invar

**Figure 7:**
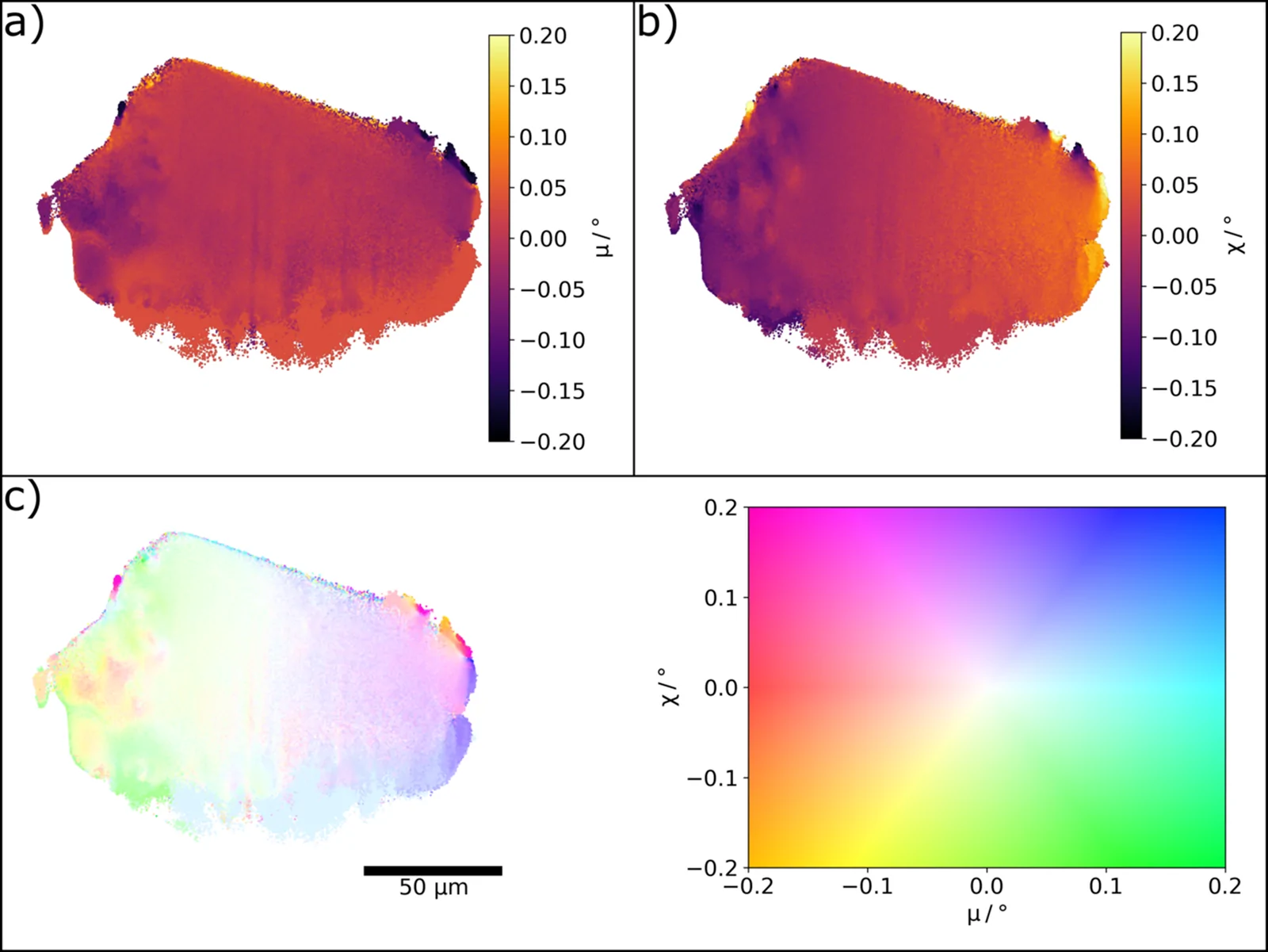
A grain within the Invar sample imaged with the diamond lenses at 33 keV. (*a*) Rocking angle μ at maximum intensity for each pixel. (*b*) Rolling angle χ at maximum intensity for each pixel. (*c*) Combined intragranular misorientation map referenced to the center of the μ and χ rocking curves. The colormap and scale bar are indicated.

An Invar alloy sample was investigated using DFXM at a photon energy of 33 keV with the diamond CRL objective. Invar is an Fe–Ni alloy known for its low thermal expansion coefficient and features relatively high X-ray attenuation compared with pure iron, making it a representative test case for thicker and compositionally heavier materials. An Fe–36Ni Invar block (composition in wt%: 0.05 C, 0.6 Mn, 0.05 S, 0.40 Si, 38 Ni, 0.5 Co, 0.1 Al, 0.25 Cr, 0.10 Mg, 0.015 P, 0.10 Ti, 0.10 Zr, balance Fe) was heat treated at 825°C for 1.5 h. Specimens were then extracted by electrical discharge machining and polished using diamond suspensions with particle sizes down to 1 µm. The material exhibits a microstructure consisting of equiaxed grains with sizes ranging from approximately 20 µm to 60 µm. The specimen has an approximately square cross section with a thickness of about 400 µm. A single grain within the equiaxed polycrystalline microstructure was selected for imaging, and intragranular mosaicity was mapped by scanning the rocking angle μ and the rolling angle χ. While the grain has a size of about 150 µm and therefore is an outlier in the grain size distribution as described before, representative electron-backscattering diffraction (EBSD) measurements of this Invar alloy confirm the existence of larger grains. Fig. 7 ▸ shows the resulting μ- and χ-dependent intensity maps together with the combined intragranular misorientation map. The magnification was *M* = 32.5 with the 2× far-field objective and the effective pixel size was 200.0 nm. In contrast to the recrystallized pure iron grain, the Invar grain exhibits a significantly larger intragranular orientation spread. It is on the order of ∼0.4°, corresponding to approximately four times the angular spread observed in the recrystallized iron case. Despite this increased spread, the microstructure remains relatively well ordered, with only a limited number of subgrains and localized defect-related features visible in the misorientation maps.

Taken together, these examples demonstrate that diamond CRL objectives enable high-quality DFXM measurements through sample thicknesses approaching 0.5 mm in iron- and nickel-based materials, which are not readily accessible at the photon energies typically employed with Be CRL optics. Operation at photon energies above 30 keV substantially reduces X-ray absorption and beam-induced heating compared with the 17 keV regime commonly used at ID03, thereby opening new experimental possibilities, including high-energy and pink-beam DFXM imaging modes (Yildirim *et al.*, 2025 ▸; La Bella *et al.*, 2025 ▸) for high-*Z* materials.

The results show that diamond CRLs are a viable alternative to common Be CRLs. At 17 keV, they exhibit a smaller FOV and slightly reduced contrast and therefore spatial resolution compared with the Be CRL currently used at ID03. In theory, Be is the superior material for basically all photon energies, but the difference in δ/β ratio gets smaller with increasing photon energy, so that the choice between diamond and beryllium is more ambiguous at 33 keV. At higher energies, the focal length of Be CRLs becomes prohibitively long for use as objective lenses. Instead, the diamond CRL is compared with an aluminium CRL at 33 keV, where it shows slightly better resolution and a larger FOV. None of the investigated lenses exhibit any relevant radial distortion.

## 4. Conclusion

Diamond CRLs for use as an objective in DFXM were characterized regarding their contrast with different resolution targets from 100 nm to 200 nm and compared with an existing Be CRL at 17 keV and with an Al CRL at 33 keV. At 17 keV, the Be CRL featured slightly better contrast and FOV. At 33 keV, the diamond CRL showed better contrast and FOV than the Al CRL. None of the CRLs exhibited significant radial distortion. For an application example and to showcase the new possibilities with an objective that works at higher photon energies such as 33 keV, a recrystallized iron sample and an Invar alloy sample were investigated with DFXM.

The use of these novel diamond CRLs opens up new possibilities for DFXM. At 33 keV, thick samples or samples containing heavy elements can now be investigated, which before exhibited too much attenuation. For example, an iron sample with a thickness of 500 µm exhibits negligible transmission at 19 keV (8.5 × 10^−6^), while for 33 keV the transmission is at least 8.6%, rendering it possible to measure the sample. With the Be CRL and its limited energy range, said iron sample had to be thinned down to 100 µm to achieve a transmission of 9.7% at 19 keV. Working at 33 keV, less sample thinning is necessary and true bulk properties of samples such as iron can be investigated (Schoonjans *et al.*, 2011 ▸). Given the limited availability of Be CRLs, diamond CRLs are a more than adequate alternative. Furthermore, diamond CRLs may also be applied as condensers for pink beam applications (Yildirim *et al.*, 2025 ▸; La Bella *et al.*, 2025 ▸).

## Figures and Tables

**Figure 6:**
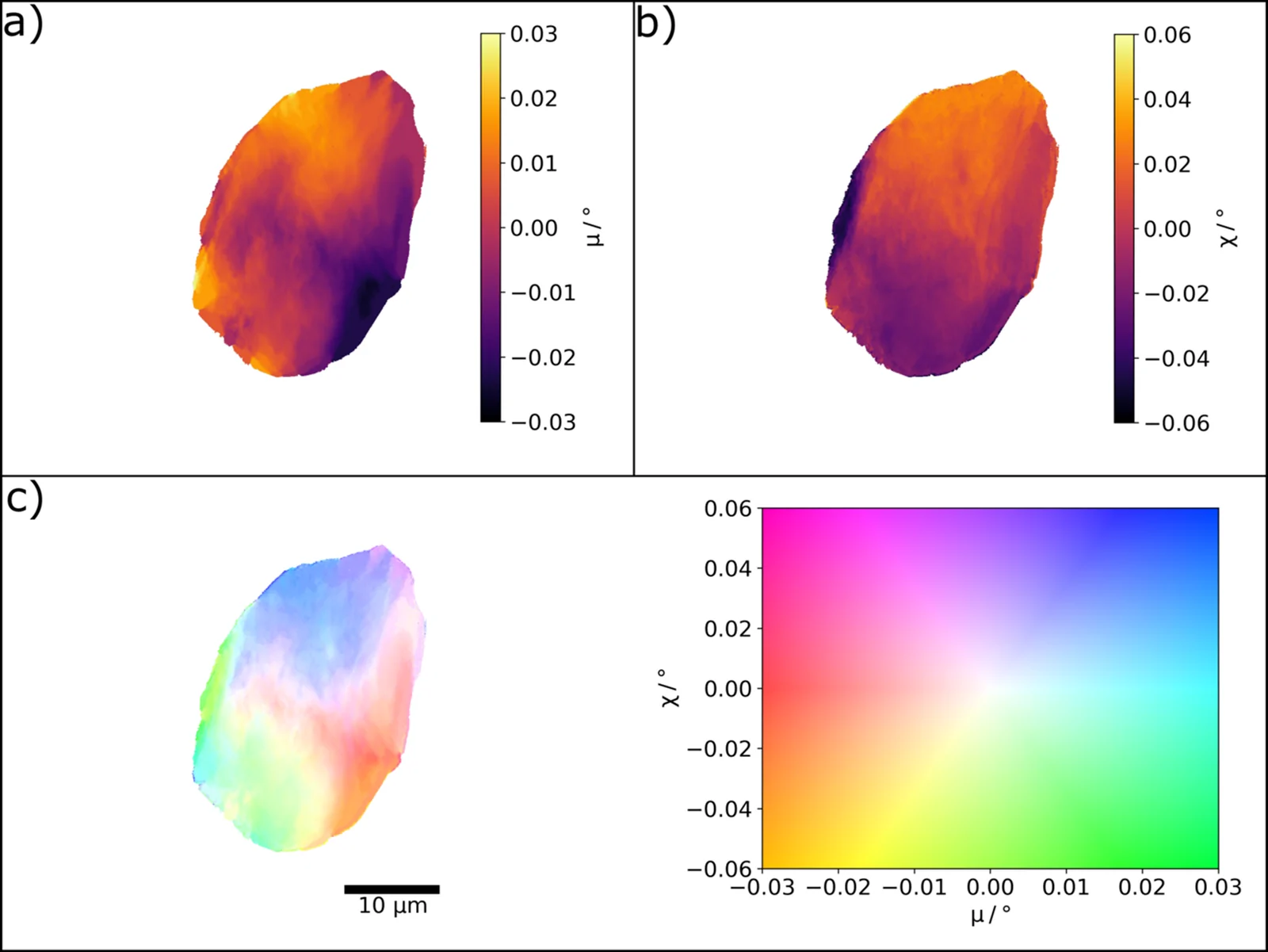
Grain 2 of the recrystallized iron sample imaged with the diamond lenses at 33 keV. (*a*) Rocking angle μ at maximum intensity for each pixel. (*b*) Rolling angle χ at maximum intensity for each pixel. (*c*) Combined intragranular misorientation map referenced to the center of the μ and χ rocking curves. The colormap and scale bar are indicated.

**Table 1:** Summary of used lens stacks (CRLs) *N* describes the number of lenses, *R* their radius of curvature at the apex, *l*_lens_ the length in the beam direction of a single lens, *A*_phys_ the diameter of the physical aperture (not accounting for absorption), and *L*_stack_ the length of the lens stack.

*E* (keV)	Name	Material	*N*	*R* (µm)	*l*_lens_ (µm)	*A*_phys_ (µm)	*L*_stack_ (mm)
17	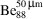	Be	88	50	1000	440	148.8
	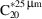	Diamond	20	25	250	120	12

33	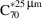	Diamond	70	25	250	120	42
	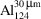	Al	124	30	600	260	148.8

**Table 2:** Horizontal and vertical Michelson contrast for the different CRLs evaluated and the maximum Michelson contrast measured for each energy The maximum Michelson contrast is given separately for each CRL at each energy, since the maximum contrast is dependent on the transmission of the CRL.

*E* (keV)	Size (µm)	Name	Vertical contrast	Horizontal contrast	Maximum contrast
17	100	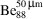	0.015 (3)	0.009 (4)	0.142 (5)
	150	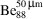	0.0561 (18)	0.068 (3)	0.142 (5)
		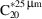	0.0082 (8)	0.0078 (8)	0.123 (4)
	200	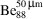	0.133 (4)	0.084 (2)	0.142 (5)
		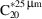	0.096 (2)	0.071 (2)	0.123 (4)

33	100	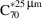	0.006 (1)	0.0034 (4)	0.054 (2)
	150	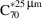	0.0331 (13)	0.0267 (19)	0.054 (2)
		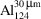	0.0158 (13)	0.0249 (13)	0.085 (4)
	200	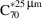	0.0630 (8)	0.0647 (6)	0.054 (2)
		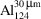	0.0448 (15)	0.0435 (13)	0.085 (4)

37	150	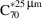	0.0163 (7)	0.0094 (3)	0.036 (1)

**Table 3:** Summary of the measurement parameters and theoretical values for the numerical apertures The effective pixel size includes the optical magnification by the objective of the detector microscope. For the measurements presented here, the 10× objective was used. The NAs of the Be and diamond CRLs are aperture-limited, while the NA of the Al CRL is absorption-limited.

*E* (keV)	Name	*M*	Effective pixel size (nm)	NA ×10^−4^
17	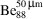	151.7	42.9	5.47
	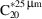	155.9	41.7	2.25

33	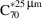	140.8	46.2	2.02
	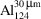	140.8	46.2	1.5

37	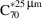	112.7	57.7	1.62

**Table 4:** Radial distortion parameters *k*_1_ for the different CRLs at different energies and their FOV The effective pixel size includes the optical magnification by the objective of the detector microscope. For the measurements at 17 keV, the 2× objective was used; for the measurements at 33 keV, the 10× objective was used.

*E* (keV)	Name	*k*_1_ (µm^−2^)	FOV (µm)	Effective pixel size (µm)
17	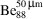	1.3 (4) × 10^−5^	125.11	212.8
	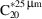	1.0 (7) × 10^−5^	91.36	227.2

33	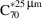	1.0 (12) × 10^−5^	89.44	45.0
	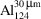	0.3 (12) × 10^−5^	64.55	45.2

**Table 5:** Summary of the characterization results for the average (horizontal and vertical) Michelson contrast for the 150 nm resolution target, the FOV and the theoretical NA for the different lenses at different energies The FOV has not been measured for the diamond CRL at 37 keV.

*E* (keV)	Name	Contrast at 150 nm	FOV (µm)	NA ×10^−4^
17	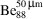	0.0621 (18)	125.11	5.47
	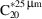	0.0080 (6)	91.36	2.25

33	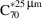	0.0299 (12)	89.44	2.02
	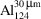	0.0204 (10)	64.55	1.5

37	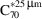	0.0129 (4)		1.62

## Appendix A Derivation of the *k*_1_ parameter

The parameter *k*_1_ was determined by calculating a histogram of the marked edge points of the checkerboard pattern in dependence on the radial distance to the center of the intensity distribution of the image. For this, all edges of the checkerboard pattern are marked by an algorithm, or, when this was not feasible, by hand. The center of mass of the intensity distribution was determined in the *x*- and *y*-direction of the image. The number of edge points *N*(*r*) is proportional to the radial distance *r*, because the area of the annulus *A*(*r*) is proportional to the radial distance as well,

Any radial distortion will influence the otherwise constant spacing between the edge points and their density and thereby *N*(*r*). This will lead to a deviation of the linear relationship between *N*(*r*) and *r*, which can be described with the Brown–Conrady radial distortion model. Specifically, the number of edge points is proportional to the following formula,

*N*(*r*) is then fitted with the following formula,

Here, *a* is a proportionality factor depending on the edge point spacing and *k*_1_ = *b*/4. A cell close to the intensity distribution center was chosen as the center of the checkerboard pattern and used to measure the size of that cell in pixels, assuming the center of a potential pin-cushion or barrel distortion to be close to the center of the intensity distribution.
